# SEBS-Grafted Itaconic Acid as Compatibilizer for Elastomer Nanocomposites Based on BaTiO_3_ Particles

**DOI:** 10.3390/polym12030643

**Published:** 2020-03-12

**Authors:** Héctor Aguilar-Bolados, Raúl Quijada, Mehrdad Yazdani-Pedram, Santiago Maldonado-Magnere, Raquel Verdejo, Miguel A. Lopez-Manchado

**Affiliations:** 1 Facultad de Ciencias Físicas y Matemáticas, Universidad de Chile, Beauchef 851, Santiago 8370456, Chile; raquijad@ing.uchile.cl; 2 Facultad de Ciencias Químicas y Farmacéuticas, Universidad de Chile, Olivos 1007, Santiago 8380544, Chile; myazdani@ciq.uchile.cl (M.Y.-P.); santiago.maldonado@usach.cl (S.M.-M.); 3 Instituto de Ciencia y Tecnología de Polímeros, ICTP-CSIC, Juan de la Cierva, 3 28006 Madrid, Spain; r.verdejo@csic.es (R.V.); lmanchado@ictp.csic.es (M.A.L.-M.)

**Keywords:** thermoplastic elastomer, barium titanate nanoparticles, compatibilizer

## Abstract

Itaconic acid (IA) is an organic acid produced by the fermentation of sugars with *aspergillus.* It has been identified as one of the top 12 building-block chemicals. Here, we report the use of IA as a possible substitute to petroleum-based compatibilizers in polymer composite. We applied this study to thermoplastic elastomers based on styrene copolymers, since they are commonly used in blends and composites. Poly(styrene-*b*-ethylene-butylene-*b*-styrene) (SEBS) was grafted with 2.6 wt.% of itaconic acid (SEBS-g-IA) prepared by a reactive melt-mixing process, and was subsequently used to prepare composites filled with BaTiO_3_.). IA was successfully grafted as demonstrated by FTIR and XRD. SEBS-g-IA composites presented better mechanical properties, achieving an increase of Young modulus up to 80% compared with the neat polymer. This was ascribed to better dispersion and compatibility with the filler. Additionally, SEBS-g-IA showed increased dielectric permittivity, i.e., showed increased polarity, which indicates that it could potentially be used as a modifier for specialized polymers.

## 1. Introduction

The versatility of thermoplastic elastomers (TPEs) arises from their dual features, i.e., they possess the processability of thermoplastics as well as the elasticity of elastomers [1,2]. Most TPEs are copolymers, having two phases: a hard and a soft segment. Among TPEs, styrene copolymers, poly(styrene-*b*-butadiene-*b*-styrene) (SBS), poly(styrene-*b*-isoprene-*b*-styrene) (SIS), and poly(styrene-*b*-ethylene-butylene-*b*-styrene) (SEBS), represent the largest category in which the styrene-rich domains serve as the hard phase [3]. SEBS is produced from the partial and selective hydrogenation of SBS, which results in good UV and heat resistance, as well as excellent mechanical properties. SEBS is usually blended with other polymers and fillers. However, its low polarity results in weak adhesion to other materials. This leads to reduction of the systems that are effectively modified by these other materials, and hinders filler dispersion [3]. Thus, improved adhesion and compatibility with specialized polymers and materials are the subject of many active research and development projects [4].

A commonly used and industrially available strategy to enhance affinity and compatibility is the inclusion of acid groups in the polymer structure. The most widely applied post-synthesis processing is the grafting reaction of maleic anhydride through reactive melt mixing [5,6,7]. Maleic anhydride (MA) is produced from the oxidation of benzene with excess air using vanadium-based catalysts [8]. SEBS grafted with maleic anhydride has been used for several purposes [9,10,11,12]. For instance, it has been used as a compatibilizing agent for composites based on polypropylene filled with wood fibers, in order to obtain enhanced interactions [11]. Likewise, it has been reported that the preparation of blends based on recycled acrylonitrile-butadiene-styrene copolymer (rABS) and SEBS-g-MA demonstrated that the presence of SEBS-g-MA improves impact performance [12]. However, current interest in nonpetroleum-based chemicals has spurred the search for possible substitutes in several fields [13,14,15]. A possible substitute for MA is itaconic acid (IA). IA is a low-cost vinyl compound with polar moieties, obtained via the fermentation of sugars with *Aspergillus terreus* and *Aspergillus itaconicus*. Consequently, itaconic acid is a renewable resource that could be useful for the synthesis of compatibilizing agents [16,17,18,19,20,21]. As such, IA has been included in the top 12 building-block chemicals by the US Department of Energy [21,22,23]. Here, we present the preparation of SEBS grafted with itaconic acid (SEBS-g-IA) and evaluate its influence in the thermal, electrical and mechanical properties in composites based on SEBS and BaTiO_3_ nanoparticles.

## 2. Experimental

### 2.1. Materials

Styrene-ethylene-butylene-styrene block copolymer (SEBS) series Calprene H6110 (30 wt.% of styrene, MW = 86.0 kg/mol) was supplied by Dynasol Elastomers (Houston, Texas, USA). Itaconic acid (IA (>98%), toluene, dicumyl peroxide (DCP) and barium titanate nanopowder (BaTiO_3_) (<100 nm) were supplied by Sigma-Aldrich (San Luis, Misuri, USA) and used as received from the suppliers.

### 2.2. Grafting of Itaconic Acid onto SEBSF

Grafting reaction of itaconic acid was carried out by using a discontinuous mixer Brabender Plasticorder Lab-station. Itaconic acid and dicumyl peroxide were mixed in SEBS at 230 °C and 75 rpm for 6 min. The resulting product was dissolved in toluene and precipitated in acetone in order to remove the unreacted itaconic acid and initiator. The extracted samples were dried before their analysis.

### 2.3. Preparation of SEBS/BaTiO_3_ Composites

In order to determine the optimum percentage of SEBS-g-IA, SEBS/SEBS-g-IA blends were prepared (Table 1). SEBS-g-IA was carefully added to a SEBS solution in toluene under stirring at 2000 rpm using a sonicator ultrasonic liquid processor (Qsonica Sonicators, Newtown, Connecticut, USA). In the case of the composite, BaTiO_3_ was mixed with SEBS-g-IA, and the suspension was added to SEBS solution in the same conditions as the blend. Finally, the samples were casted and left to dry at room temperature under a laminar air flow. The composition of the different composites is shown in Table 1.

### 2.4. Characterization

The evidence of grafting and an estimation of its extent were determined by Fourier transform infrared (FTIR) spectroscopy using a Perkin-Elmer Spectrum One spectrophotometer. The data were collected from 4000 to 700 cm^−1^ at a resolution of 4 cm^−1^ coadding 4 scans per spectra.

X-ray diffraction analysis was carried out using a Bruker D8 Advance diffractometer with a radiation source of CuK and wavelength *λ* = 1.5406 Å, with a power supply 40 kV and 40 mA. The incidence angle, *θ,* was fixed between 10° and 40° and the scan rate was 0.02°/s. The data were fitted using a Lorentz function in order to determine the solid mesophase contributions. The interplanar distance of solid mesophases was calculated by applying Bragg’s law:

(1) $$d=\frac{\lambda }{2\mathrm{sen}\;\theta }$$

where *d* was the interlayer distance and *θ* was the angle of the reflection plane. Likewise, the mesophase size, *D,* was determined using Debye–Scherrer equation:

(2) $$D=\frac{K\lambda }{\beta \cos\theta }$$

where *K* was the shape factor (*K* = 0.9) and *β* was the line broadening at half the maximum intensity (FWHM).

Thermogravimetric (TGA) analysis was performed using a Netzsch thermogravimetric analyzer model Iris TG 209 F1. The temperature program was run from 25 to 800 °C at a heating rate of 5 °C/min in nitrogen atmosphere.

The tensile tests were carried out using an Instron dynamometer model 3366 at room temperature. The measurements were carried out at a crosshead speed of 200 mm/min^−1^ and a distance between clamps of 20 mm, according to the ASTM D412 specifications. Dog-bone-shaped specimens were cut from the membranes, having 35 mm length and 0.5 mm thickness. An average of five measurements for each sample was recorded.

The morphology of the samples was studied using high resolution scanning electron microscopy, Inspect F50.

Dielectric properties were performed on a broadband dielectric spectrometer model BDS-40, Novocontrol Technologies, GmbH, over a frequency range of 10^−1^ to 10^6^ Hz at room temperature. Thin film disc-shaped samples were held in the dielectric cell between two parallel gold-plated electrodes. The amplitude of the AC electric signal applied was 1 V.

## 3. Results and Discussion

### 3.1. Grafting of Itaconic Acid onto SEBS

**Figure 1:**
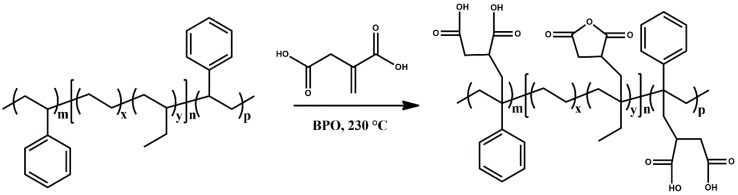
Scheme of grafting reaction of itaconic acid onto SEBS.

The functionalization of SEBS with itaconic acid was carried out by a reactive melt-mixing process in the presence of dicumyl peroxide, which is a chain-growth polymerization initiator. The initiator provided free radicals, which reacted with abstracting tertiary hydrogen atoms present in SEBS’s backbone [24]. Macroradical species can be stabilized in tertiary carbon atoms, by inductive effects in ethylene/butylene segments and mesomeric effects in polystyrene segments. The polymer radical sites can react with the vinyl function of itaconic acid. Subsequently, the itaconic function can exhibit a dehydration reaction, generating five-membered anhydride rings as seen in Figure 1 [25].

**Figure 2:**
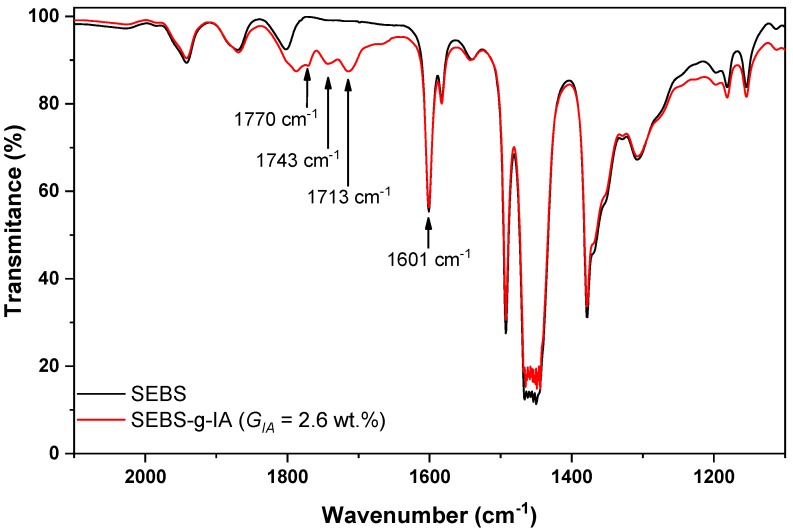
FTIR spectra of SEBS and SEBS grafted with 2.6 wt.% of itaconic acid.

Figure 2 shows the FTIR spectra of SEBS and SEBS-g-IA. The grafting reaction gave rise to three new absorption bands, at 1713 and 1743 cm^−1^, associated with the carbonyl stretching of the carboxylic acids of the itaconic acid [25,26,27] and at 1770 cm^−1^ associated with a five-membered anhydride ring [25]. The grafting reaction at high temperatures promoted the dehydration of itaconic acid giving rise to the formation of itaconic anhydride [25]. In addition, both spectra showed an absorption band at 1601 cm^−1^ associated with the C=C stretching of benzene ring of styrene moieties [28].

**Figure 3:**
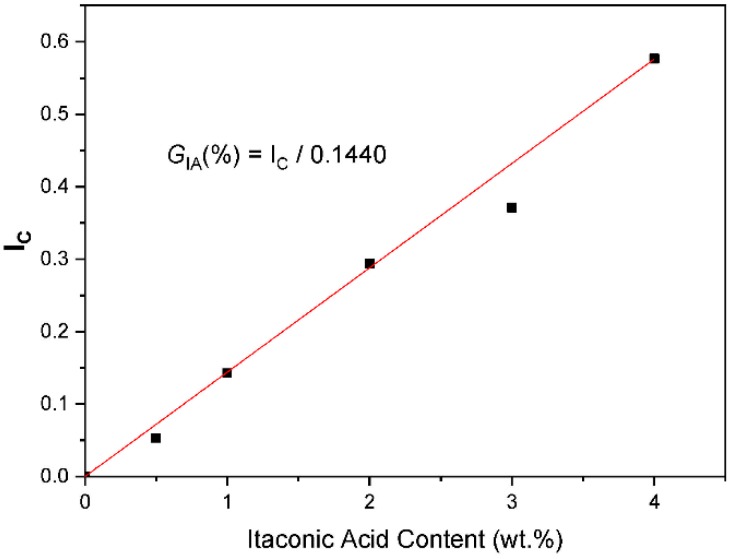
Calibration curve for estimating the percentage of grafted IA onto SEBS. Solid squares correspond to experimental data, while the red line is the fit curve.

The estimation of the percentage of grafting of itaconic acid onto SEBS was carried out by first establishing the so-called carbonyl index (*I_c_*), which is based on the Beer-Lambert law. For this, a calibration curve was built, by mixing different amounts of itaconic acid between 0.5 and 4.0 wt.% with SEBS by melt-mixing process. As shown in Equation (3), *I_c_* was estimated by considering the ratio between the integration areas of carbonyl absorption bands at 1770, 1743 and 1713 cm^−1^ and the C=C absorption band of styrene at 1601 cm^−1^ (Figure 2). The integration areas of each absorption band were determined fitting to Lorentzian-type curves. The contributions of carbonyl groups of anhydride (1700 cm^−1^) and carboxylic acid (1713 and 1743 cm^−1^) can be considered as the total percentage of itaconic acid grafted onto SEBS. Therefore, the percentage of itaconic acid grafted onto SEBS, *G_IA_* (wt.%), can be estimated using Equation (4), where the slope (1/0.1440) was determined experimentally by linear regression using the calibration curve displayed in Figure 3.

(3) $$I_{c}=\frac{\left(A_{1713}+A_{1743}+A_{1770}\right)}{A_{1601}}$$

(4) $$G_{IA}\left(wt.\%\right)=\frac{I_{C}}{0.1440}$$

**Figure 4:**
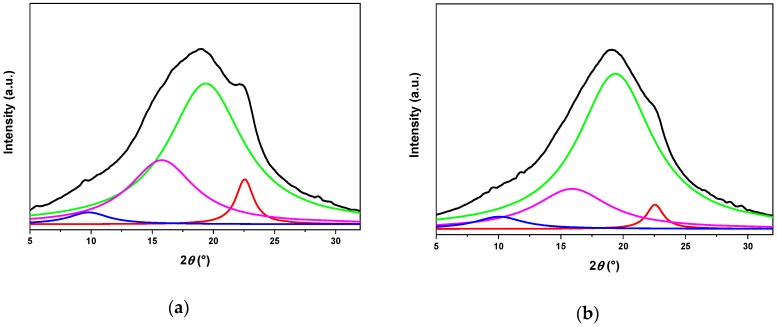
X-ray diffraction analysis of SEBS (**a**) and SEBS-g-IA (**b**), including their curve-fit. The experimental curve is the black line, while the blue, green, red and fuchsia lines correspond to the fitting curves.

SEBS grafted with 2.6 wt.% of IA (SEBS-g-IA) was selected as compatibilizing after solubility tests were carried out in toluene and carbon tetrachloride. At this grafting content, SEBS-g-IA presented a good solubility compared to those with higher contents. It is known that SEBS is composed of hard and soft domains, where the hard segments are the polystyrene blocks and the soft segments correspond to the ethylene/butylene blocks. Both soft and hard segments form solid polymer mesophases. To study the influence of the grafting on the structure of solid mesophases, both samples were analyzed by X-ray diffraction (Figure 4). The peaks were deconvoluted and the reflexion angles, the interplanar distance and the size of solid mesophases are reported in Table 2. SEBS exhibited solid mesophase contributions associated with the presence of complex polymorphs of *α*, *β* and *δ* forms of syndiotactic polystyrene phase [29,30,31,32]. The grafting of itaconic acid affected the crystalline order of styrenic segments, shifting the reflection planes at higher angles and decreasing the intensity of the peaks. In addition, the itaconic acid facilitated the formation of polar interactions between polymer chains, giving rise to a material with more rigid domains. This rigidity was ascribed to the presence of carboxylic acid end-groups present in grafted moieties of itaconic acid and the polymer backbones. The interaction between these carboxylic groups, through hydrogen bonding, inhibited the motion of the polymer chains, and thus enhanced the rigidity of the material.

**Figure 5:**
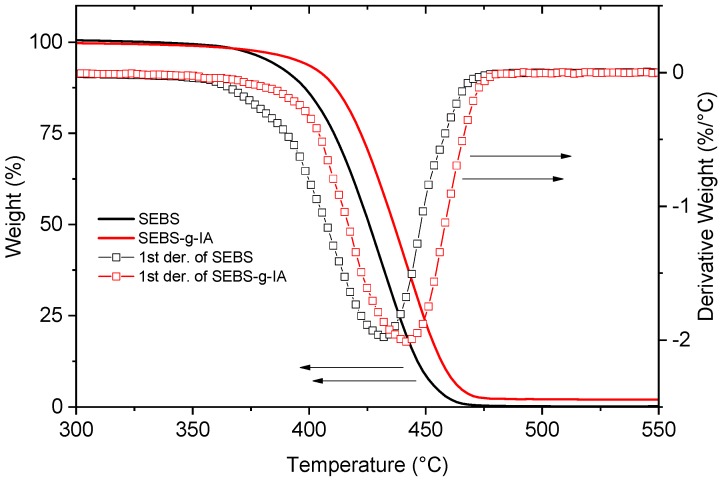
Thermogravimetric analyses of SEBS and SEBS-g-IA and the first derivative curves.

Figure 5 shows the thermogravimetric analysis and the first derivative curves of SEBS and SEBS-g-IA. SEBS remained stable up to 400 °C. Above this temperature, a single rapid decomposition process took place at 432 °C with a rate of change in weight loss of −1.98 g/°C. The grafting of itaconic acid shifted the thermal decomposition process towards the higher temperature of 441 °C. However, SEBS-g-IA showed a higher rate of change (−2.013 g/°C) than neat SEBS. The shift of the decomposition curve to higher temperatures indicated an increase of the thermal stability of SEBS. This could be promoted by the grafted itaconic acid moieties onto polystyrene segments. In this regard, the possible functionalization of polystyrene segments could be associated with the increase of thermal stability, since Tan et al. reported that the grafting of polystyrene with polar molecules, such as acrylamide, shifts the thermal decomposition of polystyrene to higher temperatures [33].

### 3.2. Properties of SEBS/BaTiO_3_ Composites Using Itaconic Acid as Compatibilizer

**Figure 6:**
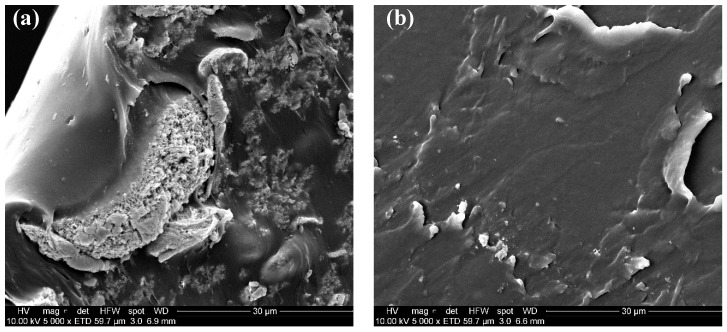
SEM images of SEBS/BaTiO_3_ 20 (**a**) and SEBS/SEBS-g-IA 60/BaTiO_3_ 20 (**b**).

**Figure 7:**
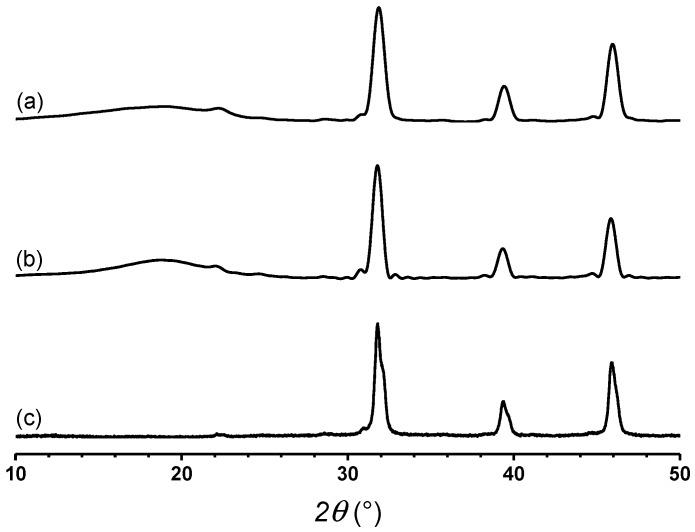
X-ray diffraction analysis of SEBS/BaTiO_3_ 20 (**a**) and SEBS/SEBS-g-IA 60/BaTiO_3_ 20 (**b**) and BaTiO_3_ (**c**).

Table 3 shows the mechanical properties of SEBS and composites SEBS/BaTiO_3_ in the presence of SEBS-g-IA as a compatibilizer. BaTiO_3_ behaved as a reinforcing agent, increasing the Young’s modulus and strength of SEBS and slightly decreasing its elongation at break. The addition of SEBS-g-IA induced a sensible increase of the mechanical properties of SEBS. This result was ascribed to the rigidity increase of the polar groups present in IA, which was also reflected in a decrease in the elongation at break of the material. Similar results have been observed by other authors when analyzing the mechanical properties of maleic anhydride grafted SEBS [4]. It is also demonstrated the effective compatibilizing effect of SEBS-g-IA for the preparation of SEBS/BatiO_3_ composites. In the presence of the compatibilizer, Young’s modulus increases by almost 80%, probably because it favors the formation of interactions between its polar moieties, such as anhydride acid and carboxylic acid, and the surface of BaTiO_3_ particles. Such increase in the matrix polarity due to IA has previously been reported in an olefinic elastomer, in particular in ethylene-propylene copolymer [25]. A further indication of the presence of these interactions is the better dispersion achieved thanks to the presence of SEBS-g-IA (Figure 6). Finally, X-ray diffraction analysis shows that SEBS/BaTiO_3_ and SEBS/SEBS-g-IA/BaTiO_3_ present characteristic peaks at 22.2°, 31.6°, 38.9° and 45.3°, corresponding to (100), (110), (111) and (200) planes of cubic BaTiO_3_ (Figure 7) [34]. The similitude observed in the diffraction patterns of both composites indicates that the mixing of BaTiO_3_ with polymer does not affect the crystallinity of nanoparticles [35].

**Figure 8:**
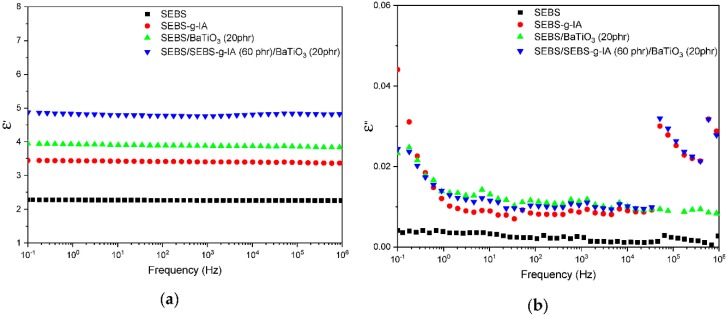
Dielectric permittivity (**a**) and dielectric loss (**b**) as function of frequency of SEBS-BaTiO_3_ composites studied.

Figure 8 shows the dielectric permittivity (8a) and dielectric loss (8b) as function of frequency in the range between 10^−1^ and 10^6^ Hz, measured at room temperature. It was observed that the addition of BaTiO_3_ increased the dielectric permittivity of SEBS, achieving a value of 3.85 at a frequency of 0.1 Hz. The presence of SEBS-g-IA further increased the dielectric permittivity to 4.85 at 0.1 Hz, which suggested that SEBS-g-IA behaved as compatibilizer by promoting the filler/polymer adhesion at the interface. This interaction promoted the interfacial polarization and, then, increased the dielectric permittivity. Similar results have been achieved by other authors using SEBS grafted with anhydride maleic (SEBS-g-MA) [36]. Other authors have observed a similar behavior when the BaTiO_3_ particles are coated with dopamine and dispersed in silicone rubber [37]. Additionally, the dielectric loss (Figure 8b) remained low with a slight increase at low frequencies due to the presence of the polar groups of the itaconic acid and the BaTiO_3_ particles. [38].

## 4. Conclusions

SEBS successfully grafted with itaconic acid showed improved mechanical and dielectric properties and thermal stability. The grafting of itaconic acid affected the solid mesophases, giving rise to a polymer with more rigid domains. In addition, SEBS-g-IA behaved as an effective compatibilizer for the preparation of SEBS/BaTiO_3_ composites. SEBS-g-IA promoted the homogeneous dispersion of BaTiO_3_ in the SEBS matrix, which resulted in a sensible increase of the mechanical properties of the composite. Moreover, SEBS-g-IA increased the dielectric permittivity without significantly varying the dielectric loss. Therefore, SEBS-g-IA could be a promising route to improve compatibility and adhesion with other specialized polymers.

## Figures and Tables

**Table 1:** Composition of poly(styrene-*b*-ethylene-butylene-*b*-styrene) (SEBS)/BaTiO_3_ composites.

Sample	SEBS (phr)	SEBS-g-IA (phr)	BaTiO_3_ (phr)
SEBS	100	0	0
SEBS/BaTiO_3_	100	0	20
SEBS/SEBS-g-IA 60	100	60	0
SEBS/SEBS-g-IA 60/BaTiO_3_	100	60	20

phr: parts per hundred of rubber.

**Table 2:** XRD data of SEBS and SEBS-g-IA.

SEBS			SEBS-g-IA		
*2ϴ* (°)	*d* (Å)	*D* (Å)	*2ϴ* (°)	*d* (Å)	*D* (Å)
9.802	9.016	19.85	10.06	8.788	17.09
15.78	5.613	12.33	15.89	5.572	11.11
19.37	4.578	10.73	19.38	4.572	10.96
22.56	3.938	45.85	22.55	3.940	48.94

**Table 3:** Mechanical properties of SEBS/BaTiO_3_ composites with and without SEBS-g-IA.

Sample	Young Modulus	E50	E100	E300	Tensile Strength	Elongation at Break
	(MPa)	(MPa)	(MPa)	(MPa)	(MPa)	(%)
SEBS	13.7 ± 1.1	1.88 ± 0.11	1.98 ± 0.11	2.65 ± 0.19	2.53 ± 0.32	609 ± 30
SEBS/BaTiO_3_	17.0 ± 1.4	1.98 ± 0.07	2.05 ± 0.07	2.74 ± 0.04	3.05 ± 0.04	555 ± 22
SEBS/SEBS-g-IA 60	20.5 ± 0.2	2.18 ± 0.02	2.33 ± 0.04	3.23 ± 0.28	3.61 ± 0.40	353 ± 49
SEBS/SEBS-g-IA 60/BaTiO_3_	30.2 ± 1.2	2.23 ± 0.04	2.37 ± 0.05	3.36 ± 0.17	3.82 ± 0.31	445 ± 31
